# Impact of low‐oxalate diet on hyperoxaluria among patients suffering from nephrolithiasis

**DOI:** 10.1002/fsn3.4088

**Published:** 2024-04-05

**Authors:** Khizra Aziz, Sana Noreen, Tabussam Tufail, Izwa Ishaq, Mohd Asif Shah

**Affiliations:** ^1^ University Institute of Diet and Nutritional Sciences The University of Lahore Lahore Pakistan; ^2^ School of Food and Biological Engineering Jiangsu University Zhenjiang China; ^3^ INTI International University Persiaran Perdana BBN Nilai Negeri Sembilan Malaysia; ^4^ Department of Economics Kabridahar University Jigjiga Somali Ethiopia; ^5^ Centre of Research Impact and Outcome, Chitkara University Institute of Engineering and Technology Chitkara University Rajpura Punjab India; ^6^ Division of Research and Development Lovely Professional University Phagwara Punjab India

**Keywords:** blood oxalate, hyperoxaluria, metabolism alteration, renal stone, urine oxalate

## Abstract

Low‐oxalate diets are useful for treating hyperoxaluria in nephrolithiasis patients. This study was unique in examining how a low‐oxalate diet in addition to a standard diet affected hyperoxaluria and renal function tests in nephrolithiasis patients. The effects of a low‐oxalate diet were analyzed by different biochemical tests, that is, anthropometric measurements, blood oxalate test, renal function test, electrolyte profile test, and 24 h urine analysis. For this purpose, 112 patients were divided into 2 groups: Group T_1_ (Conventional diet) and Group T_2_ (Low‐Oxalate diet) for 8 weeks. Each group was tested at the initiation and end of the study. Using SPSS, the obtained data from each parameter were statistically analyzed. The results showed that a low‐oxalate diet had a positive effect on patients suffering from nephrolithiasis. Furthermore, after treatment, anthropometric measurement weight (kg) among the control group (T_1_) was 100.45 ± 5.65 and the treatment group (T_2_) was 79.71 ± 9.48 kg. The effect of low‐oxalate diet on renal function test: creatinine (g/d) among T_1_ was 2.08 ± 0.86 and T_2_ was 1.17 ± 0.13, uric acid(mg/d) among T_1_ was 437.04 ± 24.20 and T_2_ was 364.61 ± 35.99, urinary oxalate (mg/d) among T_1_ was 76.84 ± 10.33 and T_2_ was 39.24 ± 1.51, respectively. Sodium (mEq/d) among T_1_ was 156.72 ± 6.37 and T_2_ was 159.84 ± 6.31, potassium (mEq/d) among T_1_ was 69.91 ± 15.37 and T_2_ was 89.21 ± 6.31, phosphorus (g/d) among T_1_ was 0.96 ± 0.07 and T_2_ was 0.34 ± 0.27, respectively. This study demonstrated that nephrolithiasis patients with hyperoxaluria benefit from low‐oxalate diets. Hyperoxaluria patients should eat a low‐oxalate diet to use oxalate without affecting metabolism and eliminate it from the kidney without stones.

## Graphical abstract

Consuming high amounts of foods which are rich in oxalate content may lead to the formation of hyperoxaluria among patients suffering from nephrolithiasis. Dietary modification is a vital component in the treatment of hyperoxaluria. Nuts(Almonds), tea, coffee, prunes, chocolate, root vegetables, rhubarb, paneer, navy beans, and spinach are some of the foods highest in oxalate in the diet causing calcium oxalate stones. Low‐oxalate diet (LOD) is a one dietary pattern which is associated with lower a risk of hyperoxaluria. This diet offers a lot of low‐oxalate fruits and vegetables, herbs and spices, cereals or grains, nuts, and seeds, a moderate amount of low‐fat dairy products and little animal protein and also promotes urinary citrate excretion, a key inhibitor of calcium oxalate stones. Those people whose are suffered from hyperoxaluria may also attain benefits from low‐oxalate diet because a low‐oxalate diet acts as a therapeutic diet and it may help in proper utilizing of oxalate without causing metabolism alteration and remove the excess oxalate from kidney without stone forming.

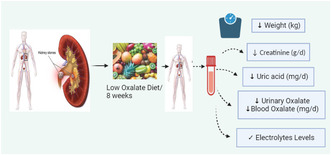

## 1 INTRODUCTION

Hyperoxaluria is one of the most widespread types of kidney disease. Oxalate excretion in the urine that is elevated or above 40 mg/dL in a 24‐hour period is known as hyperoxaluria (Vieira et al., 2020). Symptoms of hyperoxaluria include renal colic (severe abdominal pain), flank pain (pain in the back), hematuria (blood in urine), obstructive uropathy (urinary tract illness), urinary tract infections, obstruction in urine passage, and hydronephrosis (Han et al., 2015; Masood et al., 2023). An estimated 5%–15% of people worldwide have hyperoxaluria, especially in men 10%, and 6% in women over their lifetime (Noori et al., 2014), whereas in Pakistan it affects 12% (Ahmad et al., 2016). There are two distinct clinical manifestations of hyperoxaluria. A metabolic disorder caused by deficient enzyme activity is known as primary hyperoxaluria (Devresse et al., 2020). Contrarily, Secondary hyperoxaluria is the result of increasing digestible oxalate consumption, oxalate precursors, or changes in the intestinal microbiota (Bhasin et al., 2015). Urine calcium oxalate supersaturation can cause kidney stones in this illness. Approximately 80% of calcium‐containing kidney stones are calcium oxalate. Oxalate is mostly excreted via the kidneys. The average healthy person excretes 10–40 mg of oxalate daily. (Devresse et al., 2020; Noori et al., 2014). Hyperoxaluria is clinical when 40–45 mg/24 hours is exceeded. Oxalate causes renal calculi most effectively. Calcium oxalate crystals grow 2.5%–3.5% when urine oxalate exceeds 40 mg. (Bhasin et al., 2015; Demoulin et al., 2022; Marengo & Romani, 2008). Primary hyperoxaluria is extremely uncommon, with a prevalence of just 0.3 per 1000,000 people, or around 1000 people in the United States. It frequently results in repeated, calcium oxalate crystals formation, kidney loss function, and intensifying kidney impairment (Monsour et al., 2015; Wyatt & Drüeke, 2020). Primary hyperoxaluria subtypes 1, 2, and 3 are rare inherited glyoxylate metabolism diseases caused by pathogenic alanine glyoxylate aminotransferase gene and glyoxalate/hydroxypyruvate reductase gene mutations. An AGT deficit causes primary hyperoxaluria type 1. Alanine glyoxylate aminotransferase, a liver‐only enzyme, regenerates glyoxylate to glycine. Alanine glyoxylate aminotransferase is a reaction‐causing agent that is only present inside the liver which catalyzes the regeneration of glyoxylate to glycine (Monsour et al., 2015). An excess oxalate and glycolate are generated as an outcome of the deficit, which causes glyoxylate to accumulate (Witting et al., 2021). It has been established that the alanine glyoxylate aminotransferase gene mutation on chromosome 2 is associated with an absence of a B6‐dependent enzyme (AGT) (Dill et al., 2022).

Dietary modification is a vital component throughout the medical management of hyperoxaluria when attempts ought to be addressed to limit oxalate consumption in the dietary regimen. Low‐oxalate diet (LOD) is a dietary pattern that has already been linked to a lower risk of hyperoxaluria. This study places a strong emphasis on the Low‐oxalate diet, which offers a lot of low‐oxalate fruits and vegetables, herbs and spices, cereals, dry fruits and seeds, an adequate quantity of little‐fat milk products, and little animal protein. People who followed Low‐oxalate diets were discovered to have lower stone formation rates in an earlier observational study (Lange et al., 2014). This diet also promotes urine citrate excretion, an important inhibitor of calcium stones because of the increased number of fruits, greens, and little quantity of meat items. The consumption of calcium ought not to be minimized because it creates associations with oxalate or limits its utilization (Gupta et al., 2021). Fluids or water intake should be 2–3 liter/day because it helps in the removal of calcium oxalate stones from the kidney. However inappropriate consumption of ascorbic acid must be discouraged. In this study, we investigated a category of individuals having a high risk of developing hyperoxaluria or calcium oxalate stones and assessed the benefits of a low‐oxalate diet on urinary lithogenic risks and urine supersaturation (Witting et al., 2021). There are some limitations associated with this study including less awareness about the low‐oxalate diet, very little data available on the low‐oxalate diet and hyperoxaluria, and inconsistency of patience in following diet. The aims of this study was to compare the impact of a low‐oxalate diet in addition to a conventional diet on hyperoxaluria among patients suffering from nephrolithiasis and its renal function test.

## 2 MATERIALS AND METHODS

The study was conducted at the Urology Department of General Hospital, Lahore, Pakistan. A purposive sampling technique was used in the study. A low‐oxalate diet plan was prepared, consisting of 1800 kcal per day. To plan the diet or prepare the sample, an inclusion and exclusion criterion was used. Included food list is mentioned in Table 1. The study comprised hyperoxaluric patients of both genders (urine oxalate content >40 mg/day) between the age range of 20–55 years, who had normal BMI. Patients with hypocitraturia and hyperuricosuria were excluded based on the predetermined criteria. Individuals with diabetes, autoimmune disorders, a history of bariatric surgery, hepatic conditions, thyroid disorders, parathyroid disorders, or immunological disorders were excluded from participation. Additionally, individuals who were currently using cholestyramine, potassium citrate, or calcium supplements were not included in the study.

**TABLE 1:** Low oxalate food items.

Fruits	Vegetables	Grains	Proteins	Dairy	Fat	Beverages	Snacks
Apple	Cauliflower	Oats	Egg	Low Fat Milk	Olive Oil	Water	Popcorn
Pear	Broccoli	Rice	Chicken	Yogurt	Butter	Herbal tea	Rice cake
Peach	Peppers	Quinoa	Turkey	Cheese		Decaffeinated	
Melons	Zucchini		Fish				
	Celery						
	Asparagus						

### 2.1 Methods of data collection

The participants who met the study inclusion criteria were enrolled and divided into two groups: the conventional diet group (T_1_) and the low‐oxalate diet group (T_2_) for 8 weeks as mentioned in Table 2. The baseline data were comprised of anthropometric measurements (weight), 24 h dietary recall, a Renal Function test, an Electrolyte profile test (Wyatt & Drüeke, 2020), Blood oxalate test (Gupta et al., 2021) and a 24 h urine test was done as mentioned by Atallah et al. (2018) The method used was authorized by the Research Ethical Committee of The University of Lahore (IRB‐UOL‐FASH/70111274/2021). All the participants signed an informed consent form. The follow‐up for patients was conducted on alternative days to ensure the intake of the planned diet for the interventional phase. All laboratory tests were done on day 1 and after 8 weeks. The baseline and post‐test study data were compared to test the study hypothesis.

**TABLE 2:** Treatment plan.

Plan	Control group T1	Treatment group T2
Diet type	Conventional diet	Low‐oxalate diet
Duration	8 weeks	8 weeks
Target group	20–55 years	

### 2.2 Statistical analysis

All the data were analyzed by using the latest version of SPSS 25. Descriptive statistics were applied for frequency distribution and were presented in the form of Mean value±Standard deviation. Baseline and post‐study results were compared by paired sample *t*‐test at *p*‐value ≤.05 level of significance.

## 3 RESULTS AND DISCUSSION

A low‐oxalate diet (LOD) is thought to be a successful dietary strategy to control hyperoxaluria and to eliminate the calcium oxalate stone among patients suffering from nephrolithiasis. This diet places a strong emphasis on foods which offers a lot of low‐oxalate fruits and vegetables, herbs and spices, cereals, dry fruits and seeds, an adequate quantity of less‐fat milk products, and little animal protein and also promotes urine citrate excretion, an important inhibitor of calcium oxalate stones. People who followed low‐oxalate diets were discovered to have lower stone formation rates in an earlier observational study (Atallah et al., 2018). A low‐oxalate diet acts as an anti‐inflammatory diet and it may help in proper utilizing of oxalate without causing metabolism alteration and remove the excess oxalate from kidney without stone forming.

### 3.1 Anthropometric measurements

**FIGURE 1:**
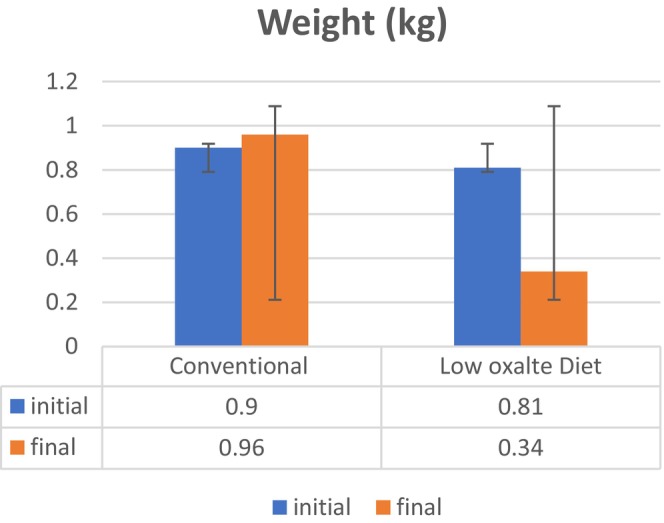
Effect of Conventional diet and low‐oxalate diet on weight (Kg) among hyperoxaluria patients suffering from nephrolithiasis.

Low‐oxalate diets consist of reducing the consumption of dietary items that are rich in oxalates (Han et al., 2015). In order to adhere to this restriction, it is advisable to prioritize the consumption of dietary items such as proteins, dairy products, white rice, as well as fruits and vegetables with low oxalate content which helps in weight reduction (Azimi et al., 2020; Siener & Metzner, 2023). Anthropometric measurements were performed at the initial and final trial day to assess impacts or effects of low‐oxalate diet among hyperoxaluria patient as shown in Figure 1. In Figure 1, the control group had an initial weight of 80.84 ± 13.93 kg, which increased to 100.45 ± 5.65 kg by week 8, while the treatment group had a baseline weight of 86.23 ± 9.70 kg and an 8th week weight of 79.71 ± 9.48 kg. As seen in Figure 1, a low‐oxalate diet reduced weight significantly (*p* = .000). Similar results were found by who mentioned that low oxalate diet is helpful in weight reduction as this diet excludes unhealthy food items and promotes healthy food choices to patients which helps in weight loss.

### 3.2 Blood oxalate levels

Oxalate is an inherent compound present in both plants (Prasad & Shivay, 2017) and animals (Whittamore & Hatch, 2017). The substance is present in some dietary sources and is also endogenously synthesized within the human body (Garrelfs et al., 2021). The majority of individuals do not necessitate apprehension regarding the presence of oxalate in their dietary intake. Nevertheless, in the event that an individual has previously had oxalate kidney stone, it is advised adhering to a low‐oxalate dietary regimen in order to mitigate the likelihood of recurring episodes of kidney stone formation, which may be highly distressing (Hennessey et al., 2019). Blood tests are employed to assess renal function and quantify oxalate concentrations in the bloodstream, for this purpose blood oxalate test was done at initial and final trial day to measure the amount of oxalate in blood which involved in causing hyperoxaluria shown in Table 3. The blood oxalate level at initial was 2.72 ± 0.99 Mg/d and on the 8th week the blood oxalate level was 2.83 ± 0.68 Mg/d among the control group and the blood oxalate level at baseline was 2.11 ± 0.52 Mg/d and on the final day was 1.43 ± 0.88 Mg/d in the treatment group. The result of this study showed significant (*p* = .000) reduction in serum oxalate level with the help of the low‐oxalate diet. Similar results were found by Gupta et al. (2021) who mentioned that a low‐oxalate diet is helpful in serum oxalate reduction. Bargagli et al., also mentioned that removing high‐oxalate content food items and encouraging healthy low‐oxalate content food choices for patients helps in maintaining a normal serum oxalate level (Bargagli et al., 2020).

**TABLE 3:** Effect of Low‐oxalate diet on serum oxalate (mg/d), Creatinine (g/d), uric acid (mg/d), and urinary oxalate (mg/d) in hyperoxaluria among patients suffering from nephrolithiasis.

Parameters	Groups		*N*	Mean ± SD	Significant *p*‐value
Blood Oxalate	T1	Initial	56	2.72 ± 0.99	.218^NS^
		Final		2.83 ± 0.68	
	T2	Initial	56	2.11 ± 0.52	.003*
		Final		1.43 ± 0.88	
Creatinine	T1	Initial	56	1.68 ± 0.36	.067^NS^
		Final		2.08 ± 0.86	
	T2	Initial	56	1.51 ± 0.32	.000**
		Final		1.17 ± 0.13	
Uric acid	T1	Initial	56	442.20 ± 34.22	.633^NS^
		Final		437.04 ± 24.20	
	T2	Initial	56	420.12 ± 47.92	.000**
		Final		364.61 ± 35.99	
Urinary Oxalate	T1	Initial	56	65.43 ± 10.29	.114^NS^
		Final		76.84 ± 10.33	
	T2	Initial	56	54.76 ± 8.63	.000**
		Final		39.24 ± 1.51	

Abbreviations: **, Highly significant; *, Significant; ^NS^, Non‐significant.

### 3.3 Creatinine levels

The kidneys perform the filtration of creatinine, a metabolic waste generated by muscular activity (Mangan et al., 2018). The blood test result has been flagged due to the presence of elevated levels of creatinine, which may indicate potential impairment in renal function (Wani & Pasha, 2021). The aforementioned condition has the potential to result in the development of chronic renal disease. For this purpose, a Renal function test was performed at the initial and final trial to evaluate the effects of a low‐oxalate diet on hyperoxaluria among patients suffering from nephrolithiasis shown in Table 3. The urinary creatinine (g/d) at initial was 1.68 ± 0.3 g/d and on the 8th week the creatinine (g/d) level was 2.08 ± 0.86 g/d among the control group and the creatinine(g/d) level at baseline was 1.51 ± 0.32 g/d and on final day was 1.17 ± 0.13 g/d in the treatment group. The result of this study showed a significant (*p* = .000) decrease in creatinine levels with the help of a low‐oxalate diet as shows in Table 3. Similar results were found by Dill et al. (2022) who mentioned that a low‐oxalate diet is helpful in the reduction of creatinine level. Low‐oxalate diet maintains the creatinine level at a normal range. Creatinine is basically a promoter of calcium oxalate stone. So this diet consists of foods having citrate content which controls the activity of creatinine and stops the formation of stones.

### 3.4 Uric acid levels

Histological analysis revealed that the presence of uric acid crystals and urate crystals initiates the development and epitaxial growth of calcium oxalate crystals (Moore et al., 2022; Stitchantrakul et al., 2006). The present in vivo investigation offers supplementary support for the notion that uric acid plays a contributory role in the development of calcium oxalate stones. For reduction of uric acid level in blood, low oxalate diet played an important role. For this observation, uric acid (mg/d) levels before and after treatment was observed. the uric acid (mg/d) level at initial was 442.20 ± 34.22 mg/d and on the 8th week the uric acid (mg/d) level was 437.04 ± 24.2 0 mg/d among the control group and the uric acid (mg/d) level at baseline was 420.12 ± 47.92 mg/d and on the final day was 364.61 ± 35.99 mg/d in treatment group as mentioned in Table 3. The result of this study showed a significant (*p* = .000) decrease in uric acid with the help of the low‐oxalate diet as shows in Table 3. Similar results were found by Witting et al. (2021) who mentioned that the low oxalate diet is helpful in decrease in uric acid level. Low‐oxalate diet maintains the uric acid level at normal range. This diet consists of foods having vitamin c content which minimizes the activity of uric acid and stops the formation of stones.

### 3.5 Urinary oxalate levels

Oxalate is an endogenous metabolic byproduct. The elimination of waste substances from the body occurs via the excretion of urine (Noreen, Rehman, et al., 2023; Saland et al., 2019). Elevated amounts of oxalate can lead to the formation of kidney stones when the excess oxalate binds with calcium. The aforementioned stones are solid concretions composed of chemical substances, which have the potential to become lodged within the urinary tract (Mitchell et al., 2019). They frequently result in significant discomfort. With the help of low oxalate in diet, urinary oxalate (mg/d) may reduce. For this reason, the present study was done to check the effect of a low‐oxalate diet on urinary oxalate. Table 3. shows that the urinary oxalate (mg/d) level at initial was 65.43 ± 10.29 mg/d, and on the 8th week, the urinary oxalate (mg/d) level was 76.86 ± 10.33 mg/d among the control group and the urinary oxalate (mg/d) level at baseline was 54.76 ± 8.63 mg/d and on the final day was 39.24 ± 1.51 mg/d in the treatment group. The result of this study showed a significant (*p* = .000) decrease in urinary oxalate with the help of a low‐oxalate diet as shown in Table 3. Similar results were found by Gupta et al. (2021) who mentioned that a low‐oxalate diet is helpful in decreasing the urinary oxalate level. This diet consists of foods having low‐oxalate content minimizing the release of excess oxalate in a urine which cannot combine with calcium to form stones.

### 3.6 Electrolyte profile

**FIGURE 2:**
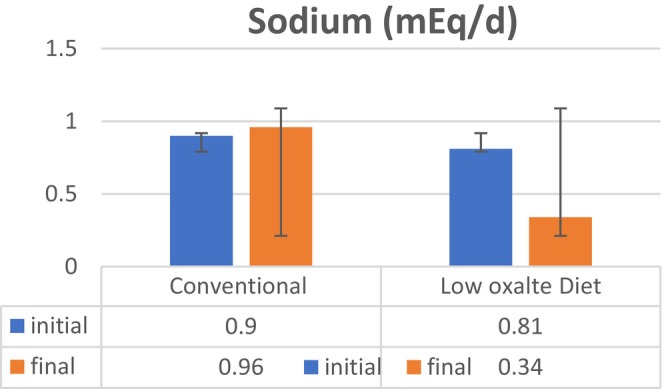
Effect of low‐oxalate diet on electrolyte profile test, that is, sodium (mEq/d) in hyperoxaluria among patients suffering from nephrolithiasis.

**FIGURE 3:**
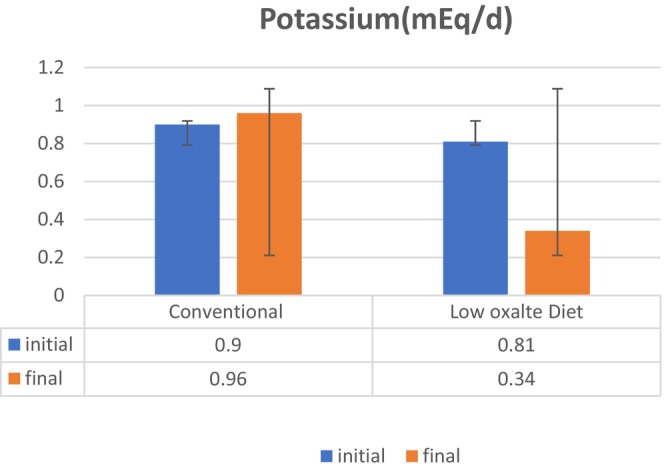
Effect of low‐oxalate diet on electrolyte profile test, that is, potassium (mEq/d) in hyperoxaluria among patients suffering from nephrolithiasis.

**FIGURE 4:**
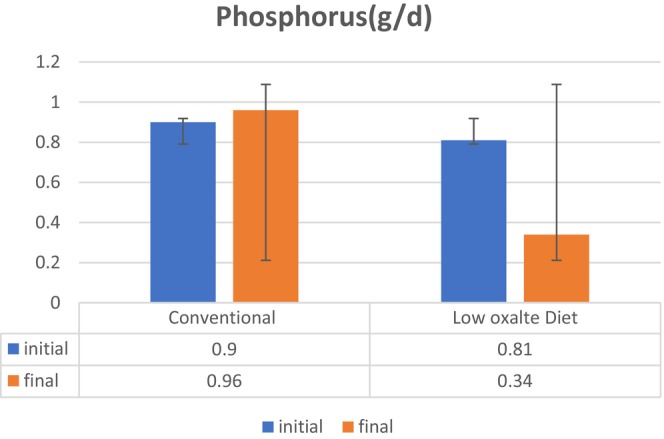
Effect of low‐oxalate diet on electrolyte profile test, that is, phosphorus (g/d) in hyperoxaluria among patients suffering from nephrolithiasis.

Kidney stones are caused by an excess of electrolytes or compounds in the urine. Because urine volume varies, electrolytes in urine are assessed by their concentration (Noreen, Khalid, et al., 2023). The higher the concentration of specific substances in the urine, the more likely the production of kidney stones (Ennis & Asplin, 2016; Ortiz‐Alvarado et al., 2011). An electrolyte profile test was performed at the initial and final trial day to evaluate the effects of the low‐oxalate diet on hyperoxaluria among patients suffering from nephrolithiasis. The sodium (mEq/d) level at initial was 142.58 ± 6.49 mEq/d, and on the 8th week, the sodium (mEq/d) level was 156.72 ± 6.37 mEq/d among the control group and the sodium (mEq/d) level at baseline was 141.05 ± 8.42 mEq/d and on the final day was 159.84 ± 6.31 mEq/d in the treatment group. The result of this study showed a significant (*p* = .000) decrease in the sodium level with the help of a low‐oxalate diet as shown in Figure 2. Similar results were found by (Kaestner et al., 2020) who mentioned that a low‐oxalate diet is helpful in decreasing sodium excretion. This diet consists of foods having low sodium along with citrate which controls the activity of sodium and stops the excess release of calcium in urine which cannot combine with oxalate to form stones. Figure 3. shows that the potassium (mEq/d) level at initial was 55.39 ± 14.49 mEq/d, and on the 8th week, the potassium (mEq/d) level was 69.91 ± 15.37 mEq/d among the control group and the potassium (mEq/d) level at baseline was 46.52 ± 8.42 mEq/d and on the final day was 89.21 ± 6.31 mEq/d in the treatment group. The result of this study showed significant (*p* = .000) improvement in potassium levels with the help of a low‐oxalate diet. Similar results were found by Azimi et al. (2020) who mentioned that a low‐oxalate diet is helpful in a decreasing potassium excretion. This diet consists of foods having moderate potassium content which stops the formation of stones by reducing the excretion of excess oxalate. Phosphorus (g/d) level at initial was 0.90 ± 0.09 g/d, and on the 8th week, phosphorus (g/d) level was 0.96 ± 0.07 g/d among the control group and phosphorus (g/d) level at baseline was 0.81 ± 0.74 g/d and on the final day was 0.34 ± 0.27 g/d in the treatment group. The result of this study showed a significant (*p* = .000) decrease in the phosphorus level with the help of a low‐oxalate diet as shown in Figure 4. Similar results were found by (Zeng et al., 2017) who mentioned that a low‐oxalate diet is helpful in decreasing phosphorus excretion. This diet consists of foods having citrate content which stops the activity of phosphorus by reducing the excretion of excess calcium in urine from bones.

## 4 CONCLUSION

Consuming high amounts of foods which are rich in oxalate content may lead to the formation of hyperoxaluria among patients suffering from nephrolithiasis. Dietary modification is a vital component in the treatment of hyperoxaluria. Nuts (Almonds), tea, coffee, prunes, chocolate, root vegetables, rhubarb, paneer, navy beans, and spinach are some of the foods highest in oxalate in the diet causing calcium oxalate stones. Low‐oxalate diet (LOD) is a one dietary pattern which is associated with lower a risk of hyperoxaluria. This diet offers a lot of low‐oxalate fruits and vegetables, herbs and spices, cereals or grains, nuts and seeds, a moderate amount of low‐fat dairy products, and little animal protein and also promotes urinary citrate excretion, a key inhibitor of calcium oxalate stones. Those people whose are suffering from hyperoxaluria may also attain benefits from a low‐oxalate diet because a low‐oxalate diet acts as a therapeutic diet and it may help in proper utilization of oxalate without causing metabolism alteration and removes the excess oxalate from kidney without any stone forming.

## AUTHOR CONTRIBUTIONS

**Khizra Aziz:** Writing – original draft (equal). **Sana Noreen:** Supervision (equal). **TABUSSAM TUFAIL:** Supervision (equal); validation (equal). **Izwa Ishaq:** Formal analysis (equal). **Mohid Asif Shah:** Writing – review and editing (equal).

## FUNDING INFORMATION

No funding was received for this study.

## CONFLICT OF INTEREST STATEMENT

The authors declare that they have no conflict of interest.

## ETHICAL APPROVAL

The study was approved by the Research Ethic Committee (REC) of The University of Lahore, Lahore, Pakistan.

## Data Availability

Data used for this study are available on request through the corresponding author, although all the relevant data have been provided here.

## References

[fsn34088-bib-0001] Ahmad, S. , Ansari, T. M. , & Shad, M. A. (2016). Prevalence of renal calculi: Type, age and gender specific in southern Punjab, Pakistan. The Professional Medical Journal, 23(4), 389–395. 10.17957/TPMJ/16.2893

[fsn34088-bib-0002] Atallah, W. , Purnell, S. , Gallante, B. , Thai, J. , & Gupta, M. J. T. J. O. U. (2018). PD17‐06 a prospective randomized controlled trial comparing the efficacy of low‐oxalate diet versus vitamin B6 and magnesium supplementation versus both in idiopathic hyperoxaluria, 199(4S), e386. 10.1016/j.juro.2018.02.968

[fsn34088-bib-0003] Azimi, T. , Eghtesadi, S. , & Abbasi, B. (2020). The comparison of major dietary patterns in people with and without calcium oxalate kidney stone: A case‐control study. Journal of Nutrition & Food Security, 5(4), 365–376. 10.18502/jnfs.v5i4.4438

[fsn34088-bib-0004] Bargagli, M. , Tio, M. C. , Waikar, S. S. , & Ferraro, P. M. (2020). Dietary oxalate intake and kidney outcomes. Nutrients, 12(9), 2673. 10.3390/nu12092673 32887293 PMC7551439

[fsn34088-bib-0005] Bhasin, B. , Ürekli, H. M. , & Atta, M. G. (2015). Primary and secondary hyperoxaluria: Understanding the enigma. World Journal of Nephrology, 4(2), 235–244. 10.5527/wjn.v4.i2.235 25949937 PMC4419133

[fsn34088-bib-0006] Demoulin, N. , Aydin, S. , Gillion, V. , Morelle, J. , & Jadoul, M. (2022). Pathophysiology and management of hyperoxaluria and oxalate nephropathy: A review. American Journal of Kidney Diseases, 79(5), 717–727. 10.1053/j.ajkd.2021.07.018 34508834

[fsn34088-bib-0007] Devresse, A. , Cochat, P. , Godefroid, N. , & Kanaan, N. (2020). Transplantation for primary hyperoxaluria type 1: Designing new strategies in the era of promising therapeutic perspectives. Kidney International Reports, 5(12), 2136–2145. 10.1016/j.ekir.2020.09.022 33305106 PMC7710835

[fsn34088-bib-0008] Dill, H. , Martin‐Higueras, C. , & Hoppe, B. (2022). Diet‐related urine collections: Assistance in categorization of hyperoxaluria. Urolithiasis, 50(2), 141–148. 10.1007/s00240-021-01290-2 34821949 PMC8956551

[fsn34088-bib-0009] Ennis, J. L. , & Asplin, J. R. (2016). The role of the 24‐h urine collection in the management of nephrolithiasis. International Journal of Surgery, 36, 633–637. 10.1016/j.ijsu.2016.11.020 27840312

[fsn34088-bib-0010] Garrelfs, S. F. , Van Harskamp, D. , Peters‐Sengers, H. , Van Den Akker, C. H. , Wanders, R. J. , Wijburg, F. A. , Van Goudoever, J. B. , Groothoff, J. W. , Schierbeek, H. , & Oosterveld, M. J. (2021). Endogenous oxalate production in primary hyperoxaluria type 1 patients. Journal of the American Society of Nephrology, 32(12), 3175–3186. 10.1681/ASN.2021060729 34686543 PMC8638398

[fsn34088-bib-0011] Gupta, M. , Gallante, B. , Bamberger, J. N. , Khusid, J. A. , Parkhomenko, E. , Chandhoke, R. , Capodice, J. , & Atallah, W. (2021). Prospective randomized evaluation of idiopathic hyperoxaluria treatments. Journal of Endourology, 35(12), 1844–1851. 10.1089/end.2021.0122 34254834

[fsn34088-bib-0012] Han, H. , Segal, A. M. , Seifter, J. L. , & Dwyer, J. T. (2015). Nutritional management of kidney stones (nephrolithiasis). Clinical Nutrition Research, 4(3), 137–152. 10.7762/cnr.2015.4.3.137 26251832 PMC4525130

[fsn34088-bib-0013] Hennessey, D. B. , Kinnear, N. , Rice, G. , Curry, D. , Woolsey, S. , & Duggan, B. (2019). Compliance in patients with dietary hyperoxaluria: A cohort study and systematic review. Asian Journal of Urology, 6(2), 200–207. 10.1016/j.ajur.2018.03.002 31061807 PMC6488745

[fsn34088-bib-0014] Kaestner, L. , Meki, S. , Moore, A. , Van Woerden, C. , & Lazarus, J. J. S. A. J. O. S. (2020). General and dietary oxalate restriction advice reduces urinary oxalate in the stone clinic setting. South African Journal of Surgery, 58(4), 210–212. 10.17159/2078-5151/2020/v58n4a3105 34096208

[fsn34088-bib-0015] Lange, J. N. , Mufarrij, P. W. , Easter, L. , Knight, J. , Holmes, R. P. , & Assimos, D. G. (2014). Fish oil supplementation and urinary oxalate excretion in normal subjects on a low‐oxalate diet. Urology, 84(4), 779–782. 10.1016/j.urology.2014.04.052 25102784 PMC4243483

[fsn34088-bib-0016] Mangan, C. , Stott, M. C. , & Dhanda, R. (2018). Renal physiology: Blood flow, glomerular filtration and plasma clearance. Anaesthesia & Intensive Care Medicine, 19(5), 254–257. 10.1016/j.mpaic.2018.02.013

[fsn34088-bib-0017] Marengo, S. R. , & Romani, A. M. (2008). Oxalate in renal stone disease: The terminal metabolite that just won't go away. Nature Clinical Practice Nephrology, 4(7), 368–377. 10.1038/ncpneph0845 18523430

[fsn34088-bib-0018] Masood, I. , Noreen, S. , Raza, K. , Khalid, W. , Rahim, M. A. , & Mohamedahmed, K. A. (2023). Effect of ketogenic diet and hypocaloric Mediterranean diet on metabolic and endocrine parameter in women suffering from polycystic ovary syndrome. International Journal of Food Properties, 26(2), 3187–3196. 10.1080/10942912.2023.2275528

[fsn34088-bib-0019] Mitchell, T. , Kumar, P. , Reddy, T. , Wood, K. D. , Knight, J. , Assimos, D. G. , & Holmes, R. P. (2019). Dietary oxalate and kidney stone formation. American Journal of Physiology. Renal Physiology, 316(3), F409–F413. 10.1152/ajprenal.00373.2018 30566003 PMC6459305

[fsn34088-bib-0020] Monsour, C. , Gregory, J. , Hatch, M. , Khan, S. , & Canales, B. (2015). Mp33‐11 calcium is more effective than vitamin B6 At reducing oxalate excretion in a gastric bypass model of hyperoxaluria. The Journal of Urology, 193(4S), e377. 10.1016/j.juro.2015.02.572

[fsn34088-bib-0021] Moore, J. P. , Mauler, D. J. , Narang, G. L. , Stern, K. L. , Humphreys, M. R. , & Keddis, M. T. (2022). Etiology, urine metabolic risk factors, and urine oxalate patterns in patients with significant hyperoxaluria and recurrent nephrolithiasis. International Urology and Nephrology, 54(11), 2819–2825. 10.1007/s11255-022-03311-4 35917078

[fsn34088-bib-0022] Noori, N. , Honarkar, E. , Goldfarb, D. S. , Kalantar‐Zadeh, K. , Taheri, M. , Shakhssalim, N. , Parvin, M. , & Basiri, A. (2014). Urinary lithogenic risk profile in recurrent stone formers with hyperoxaluria: A randomized controlled trial comparing DASH (dietary approaches to stop hypertension)‐style and low‐oxalate diets. American Journal of Kidney Diseases, 63(3), 456–463. 10.1053/j.ajkd.2013.11.022 24560157

[fsn34088-bib-0023] Noreen, S. , Khalid, W. , Khan Niazi, M. , Javed, M. , Aziz, A. , Raza, A. , Khalid, M. Z. , & Chamba, M. V. M. (2023). Application of radish pods in households and effect of their active components against different diseases: A review. International Journal of Food Properties, 26(1), 2039–2054. 10.1080/10942912.2023.2241662

[fsn34088-bib-0024] Noreen, S. , Rehman, H. U. , Tufail, T. , Badar Ul Ain, H. , & Awuchi, C. G. (2023). Secoisolariciresinol diglucoside and anethole ameliorate lipid abnormalities, oxidative injury, hypercholesterolemia, heart, and liver conditions. Food Science & Nutrition, 11, 2620–2630. 10.1002/fsn3.3250 37324915 PMC10261738

[fsn34088-bib-0025] Ortiz‐Alvarado, O. , Miyaoka, R. , Kriedberg, C. , Moeding, A. , Stessman, M. , & Monga, M. (2011). Pyridoxine and dietary counseling for the management of idiopathic hyperoxaluria in stone‐forming patients. Urology, 77(5), 1054–1058. 10.1016/j.urology.2010.08.002 21334732

[fsn34088-bib-0026] Prasad, R. , & Shivay, Y. S. (2017). Oxalic acid/oxalates in plants: From self‐defence to phytoremediation. Current Science, 25, 1665–1667. 10.18520/cs/v112/i08/1665-1667

[fsn34088-bib-0027] Saland, J. M. , Kupferman, J. C. , Pierce, C. B. , Flynn, J. T. , Mitsnefes, M. M. , Warady, B. A. , & Furth, S. L. (2019). Change in dyslipidemia with declining glomerular filtration rate and increasing proteinuria in children with CKD. Clinical Journal of the American Society of Nephrology: CJASN, 14(12), 1711–1718. 10.2215/CJN.03110319 31712386 PMC6895497

[fsn34088-bib-0028] Siener, R. , & Metzner, C. (2023). Dietary weight loss strategies for kidney stone patients. World Journal of Urology, 41(5), 1221–1228. 10.1007/s00345-022-04268-w 36593299 PMC10188387

[fsn34088-bib-0029] Stitchantrakul, W. , Sopassathit, W. , Prapaipanich, S. , & Domrongkitchaiporn, S. (2006). Effects of calcium supplements on the risk of renal stone formation in a population with low oxalate intake. Journal of Urology, 175(5), 1749. 10.1111/j.1523-1755.2004.00587.x 15916110

[fsn34088-bib-0030] Vieira, M. S. , Francisco, P. d. C. , Hallal, A. L. L. , Penido, M. G. M. , & Bresolin, N. L. (2020). Association between dietary pattern and metabolic disorders in children and adolescents with urolithiasis. Jornal de Pediatria, 96, 333–340. 10.1016/j.jped.2018.11.008 30731051 PMC9432078

[fsn34088-bib-0031] Wani, N. , & Pasha, T. (2021). Laboratory tests of renal function. Anaesthesia & Intensive Care Medicine, 22(7), 393–397. 10.1016/j.mpaic.2021.05.010

[fsn34088-bib-0032] Whittamore, J. M. , & Hatch, M. (2017). The role of intestinal oxalate transport in hyperoxaluria and the formation of kidney stones in animals and man. Urolithiasis, 45, 89–108. 10.1007/s00240-016-0952-z 27913853 PMC5358548

[fsn34088-bib-0033] Witting, C. , Langman, C. B. , Assimos, D. , Baum, M. A. , Kausz, A. , Milliner, D. , Tasian, G. , Worcester, E. , Allain, M. , & West, M. (2021). Pathophysiology and treatment of enteric hyperoxaluria. Clinical Journal of the American Society of Nephrology: CJASN, 16(3), 487–495. 10.2215/CJN.08000520 32900691 PMC8011014

[fsn34088-bib-0034] Wyatt, C. M. , & Drüeke, T. B. (2020). Stiripentol for the treatment of primary hyperoxaluria and calcium oxalate nephropathy. Kidney International, 97(1), 17–19. 10.1016/j.kint.2019.06.011 31451300

[fsn34088-bib-0035] Zeng, G. , Mai, Z. , Xia, S. , Wang, Z. , Zhang, K. , Wang, L. , Long, Y. , Ma, J. , Li, Y. , & Wan, S. P. J. B. i. (2017). Prevalence of kidney stones in China: An ultrasonography based cross‐sectional study. BJU International, 120(1), 109–116. 10.1111/bju.13828 28236332

